# Role of Madden–Julian Oscillation in predicting the 2020 East Asian summer precipitation in subseasonal-to-seasonal models

**DOI:** 10.1038/s41598-024-51506-9

**Published:** 2024-01-09

**Authors:** Jieun Wie, Jinhee Kang, Byung-Kwon Moon

**Affiliations:** Division of Science Education, Institute of Fusion Science, Jeonbuk National University, Jeonju, 54896 South Korea

**Keywords:** Climate sciences, Atmospheric science

## Abstract

The 2020 summer monsoon season in East Asia was unusually long and intense, and the Madden–Julian Oscillation (MJO) has been proposed as an underlying reason. This study analyzes the role of the MJO in the 2020 East Asian precipitation forecasts of the subseasonal-to-seasonal (S2S) model. The S2S models underestimated the cumulative precipitation over East Asia, and the models with good forecast performance yielded a distinct precipitation band over East Asia and a western pacific subtropical high (WPSH) during the analysis period. East Asian precipitation forecast performance was more closely related to the location of the center than the strength of the WPSH, with precipitation increasing with a decrease in the latitude at the center. MJO Phases 1–3 activation intensified the WPSH and shifted the center of WPSH to lower latitudes. Our results confirm that the strong East Asian precipitation in summer 2020 was partly due to changes in the characteristics of the MJO and indicate the importance of accurately estimating the MJO-WPSH coupling for reliable East Asian precipitation forecasts.

## Introduction

In 2020, the East Asian region experienced extreme precipitation, particularly the Yangtze River region of China in June, and eastern China, the Korean Peninsula, and southern Japan in July. The variation in precipitation zones during the summer is attributed to the changes in the position of the precipitation band on its northern boundary in response to the gradual movement of the western Pacific subtropical high (WPSH) northward from low-latitude regions. In 2020, the northward movement of the WPSH was particularly slower than usual, resulting in a prolonged period of precipitation that lasted until early August in the region. This amount of precipitation had adverse socioeconomic effects such as reduced agricultural production, and inconvenience to daily life. To reduce these damages, accurate forecasts of precipitation band locations and precipitation intensity are crucial.

The formation of the East Asian precipitation band is influenced by the formation of the WPSH at low latitudes, Atlantic teleconnection, blocking at mid- and high-latitudes, and the polar regions^1–3^. The WPSH is adjacent to the East Asian precipitation region, which directly affects the location of precipitation bands and precipitation intensity^4–7^. Therefore, understanding the fluctuation pattern of the WPSH is important for forecasting precipitation in East Asia. When the WPSH is well developed, it is longitudinally elongated, and when it extends westward, as it did in 2020, its western edge reaches the South China Sea region^8,9^. When warm and moist air is supplied to eastern China, the Korean Peninsula, and southern Japan along the edge of the high, precipitation increases in the precipitation band at the northern edge of the high.

The intraseasonal fluctuations of the WPSH are influenced by sea surface temperatures in the western Pacific and the Madden–Julian Oscillation (MJO)^10–13^. WPSHs have oscillations spanning 10–30 days or > 40 days^12^. The development of the WPSH leads to the development of downdrafts and the dissipation of clouds. This increases the incident solar radiation, consequently increasing the sea surface temperature and influencing the stability of the adjacent atmosphere, which can attenuate the WPSH pressure^13^. In addition, the MJO is a strong convection in the tropics with a 30–60-day cycle, and when this convection emerges in the Indian Ocean during MJO Phases 1–3, it induces a teleconnection downdraft in the WPSH region, which reinforces the WPSH pressure. At this point, the high expands to the west, resulting in the development of a thick and elongated precipitation band, which can lead to prolonged periods of intense precipitation. Supplementary Figure S1 shows that the number of active days in the month of July in MJO Phases 1–3 for the period 1991–2020 is positively correlated with both WPSH intensity and East Asian precipitation. Notably, 2020 has the second highest number of active days in MJO Phases 1–3 during the analysis period, with high WPSH intensity and East Asian precipitation. The WPSH also exhibits interannual variability, which is influenced by the El Niño-Southern Oscillation, the Pacific Decadal Oscillation (PDO), changes in the Hadley circulation, and the North Atlantic Oscillation (NAO)^14–21^.

Therefore, the WPSH exhibits a wide range of intraseasonal and interannual variability. During June–July 2020, the MJO was particularly active in Phases 1–3, leading to the emergence of strong convection over the Indian Ocean, which facilitated the development of a strong WPSH. Particularly in June, the MJO remained in phases 1–2, resulting in strong precipitation over southern China^22,23^, and in July, phases 2–3 were associated with precipitation over China, the Korean Peninsula, and the Japanese archipelago^24^. Additionally, the slow northward movement of the WPSH in 2020 that resulted in a long-term heavy precipitation event over East Asia was unusual^25^.

Subseasonal to seasonal forecasting projects provide 30 to 60 days of model forecasts to bridge the gap between weather and seasonal forecasts^26^. During this time period, the initial conditions play a crucial role, and it is also too short for the effects of boundary fields to take effect, thereby complicating the calibration of model forecasts. However, phenomena such as MJOs^27,28^, snowfall^29,30^, stratospheric-tropospheric interactions^31–33^, and extreme weather^34–37^, which span a few days to a few weeks, are crucial for weather prediction. S2S models tended to simulate weaker MJO strength and slower eastward propagation, and the amplitude of the MJO teleconnection pattern was weak^38^. In the western Pacific, the good models with a realistic representation of the atmosphere–ocean interaction were those with a small Maritime Continent barrier effect, resulting in continuous MJO propagation^39^. In this study, we evaluate the forecast performance of the 2020 East Asian summer precipitation using the S2S model and analyze the role of the MJO in the model's forecast performance.

## Results and discussion

### Precipitation prediction performance of subseasonal-to-seasonal forecasts

**Figure 1:**
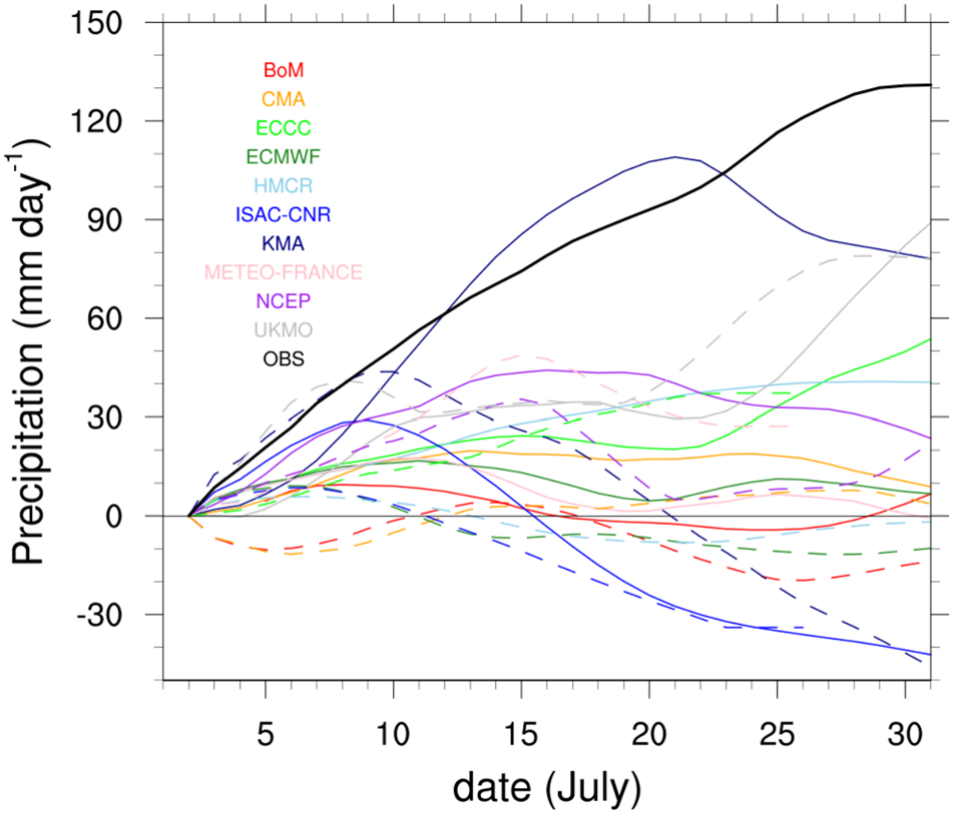
Timeseries of cumulative precipitation anomalies averaged over East Asian region of 30°N–40°N, 110–145°E in the S2S models and observation during the forecast time of July 3–30, 2020. The dashed and solid lines indicate the initialization dates June 25 and July 2, respectively. The colors of each model are shown in the figure.

Figure 1 shows the time series of cumulative precipitation over East Asia from July 3 to July 30, 2020 based on the S2S model results and observations. In general, the longer the prediction period, the worse the forecast performance; however, in this case, the performance varies depending on the prediction period because the analysis area is narrow. Therefore, we selected the initial dates of June 25 and July 2 to increase the number of analysis cases. The observation shows a steady increase during July. The S2S models generally underestimated the cumulative precipitation compared to the observations. KMA, UKMO, ECCC, and HMCR on the initialization date July 2 and UKMO, ECCC, and METEO-FRANCE on the initialization date June 25 exhibit small model errors around July 25. For negative values of cumulative precipitation, the model errors for ISAC-CNR and BoM on July 2, and ISAC-CNR, CMA, KMA, ECMWF, and HMCR on June 25 were considerable. Based on this, UKMO and ECCC exhibited small errors in their East Asian precipitation forecasts, while ISAC-CNR and CMA exhibited huge errors. HMCR and KMA yielded more optimized forecast performance for relatively short forecast periods, as their errors were drastically reduced when the initialization date was July 2 compared to when it was June 25.

**Figure 2:**
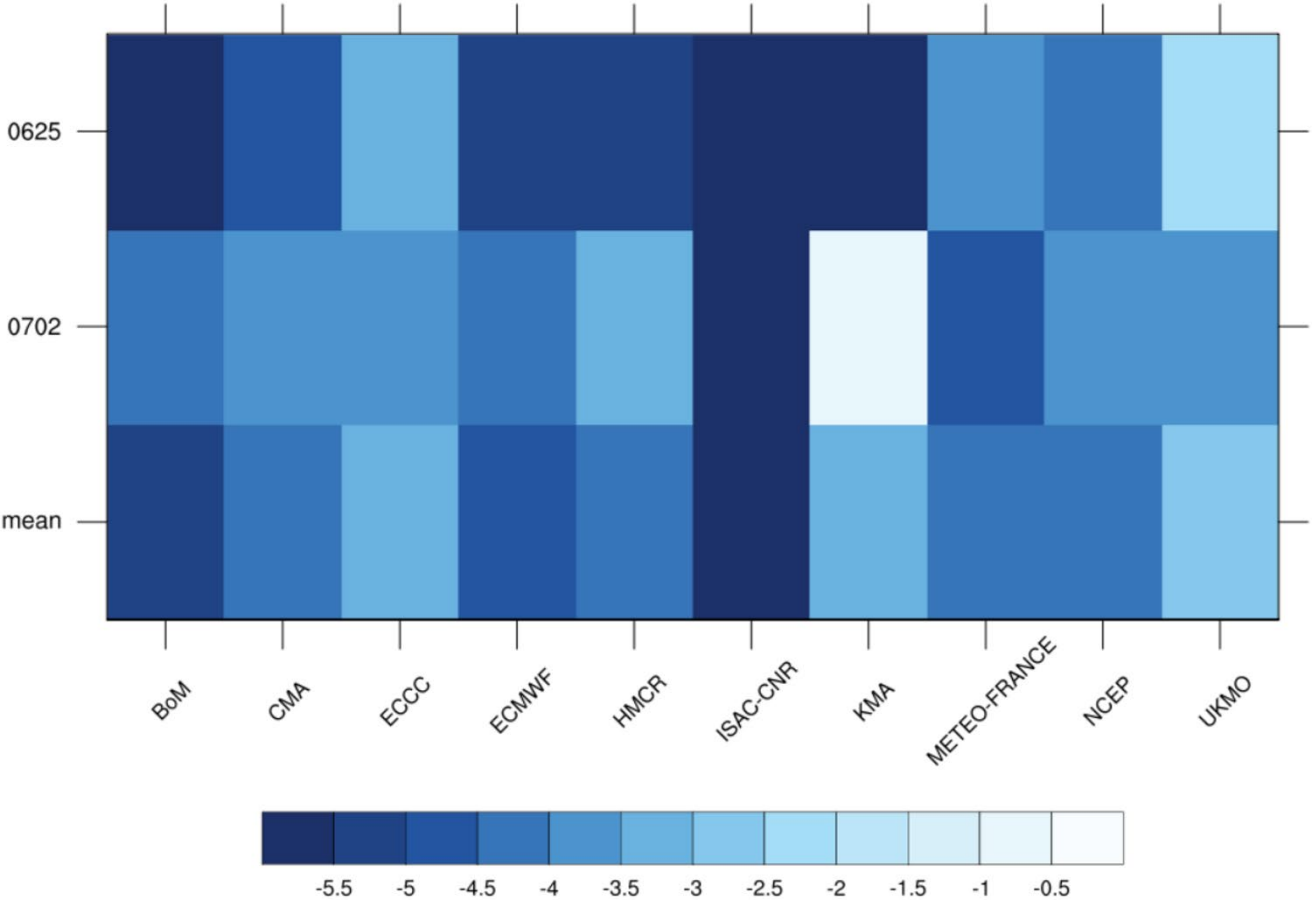
Mean bias error of East Asian precipitation for the initialization dates June 25 and July 2, and the mean initialization dates in the S2S models.

To quantitatively verify the performance of the S2S model in forecasting precipitation in East Asia, we performed a mean bias error (MBE) analysis (Fig. 2). For all the initialization dates, all models yielded negative MBE values, indicating that the models underestimated East Asian precipitation. ECCC, UKMO, and KMA yielded small errors on the initialization dates of June 25 and July 2. ISAC-CNR yielded a huge error with an MBE value of > -5.6, and BoM, ECMWF, HMCR, and KMA yielded huge errors on the initialization date June 25. Evidently, the error increased as the forecast period increased. Considering the aforementioned dates, ECCC, KMA, and UKMO performed remarkably, while ISAC-CNR, BoM, and ECMWF performed poorly.

**Figure 3:**
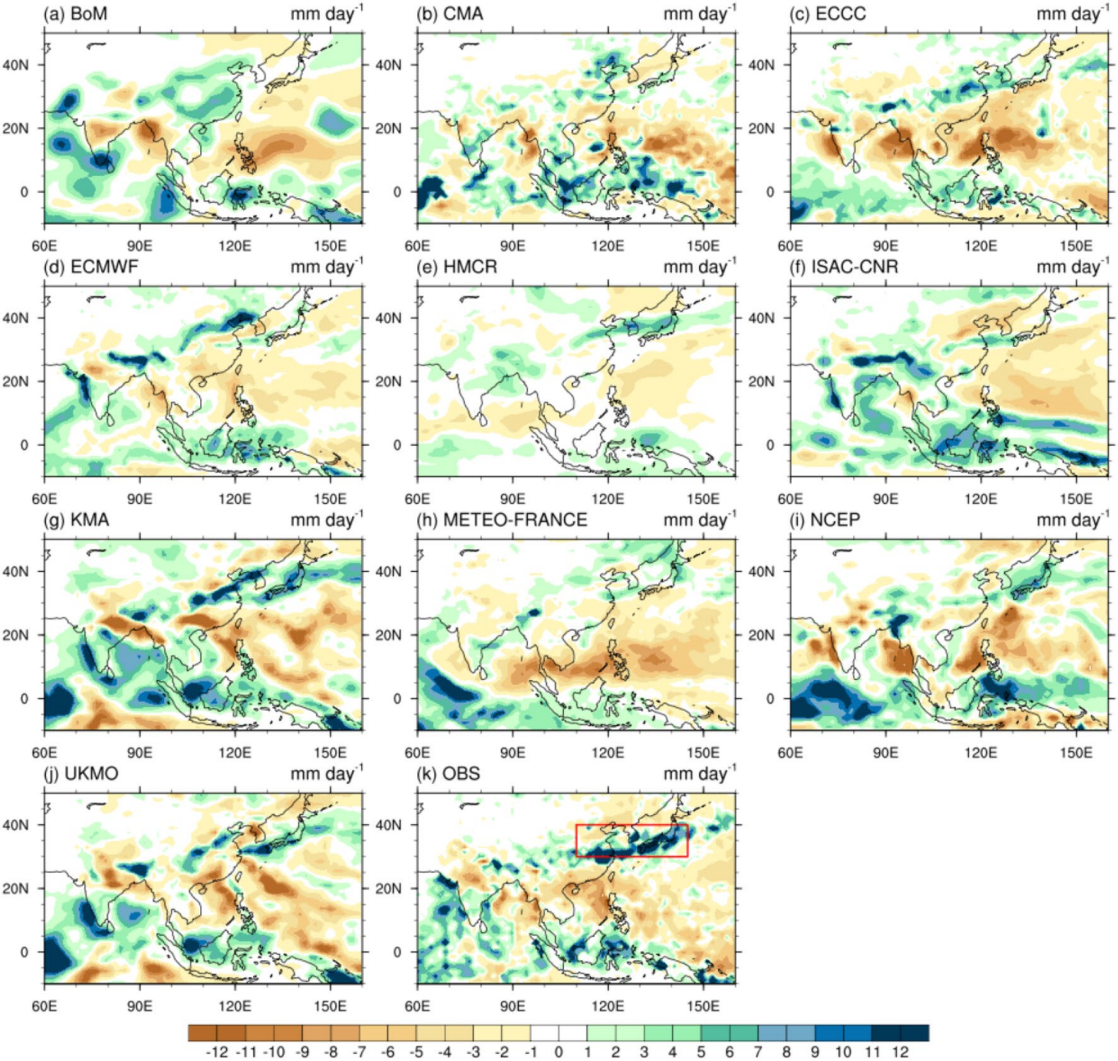
Distribution of precipitation anomalies (mm day^−1^) for the initialization date July 2 in (**a**) BoM, (**b**) CMA, (**c**) ECCC, (**d**) ECMWF, (**e**) HMCR, (**f**) ISAC-CNR, (**g**) KMA, (**h**) METEO-FRANCE, (**i**) NCEP, (**j**) UKMO, and (**k**) observations during the forecast period of July 3–25, 2020. The red box in (**k**) indicates the East Asian region in this study.

Figure 3 shows the precipitation anomaly distribution of the 10 S2S models on the initialization date July 2. Figure 3k shows an analysis of the observations, and the red box indicates the study area. The July 2020 East Asian precipitation band shows a distinct line across eastern China, the southern Korean Peninsula, and southern and central Japan. Large areas of dryness without precipitation were observed in the WPSH area, which is located over a large area of ocean bordering a distinct band of precipitation over East Asia. For ECCC, HMCR, KMA, NCEP, and UKMO, which yielded reasonable precipitation forecasts for July 2, the precipitation bands were weaker than the observations but had a distinct shape. The dry zones in the WPSH region were well distributed. The poor precipitation prediction performance of ISAC-CNR and METEO-FRANCE was attributed to the weak precipitation bands in East Asia, while BoM and ECMWF were biased towards inland China. In the case of CMA, which exhibited a similar prediction performance to that of ECCC, precipitation bands did not appear, and areas with high precipitation were not included in the study area. For the initialization date June 25, the models with good forecast performance, ECCC, METEO-FRANCE, and UKMO, showed clear precipitation band shapes (Supplementary Fig. S2). The models with poor forecast performance, ECMWF, HMCR, ISAC-CNR, and KMA, exhibited weak precipitation bands. BoM had a precipitation band outside the study area. NCEP showed the precipitation zone concentrated on the Korean Peninsula, but not in the form of a precipitation band.

**Figure 4:**
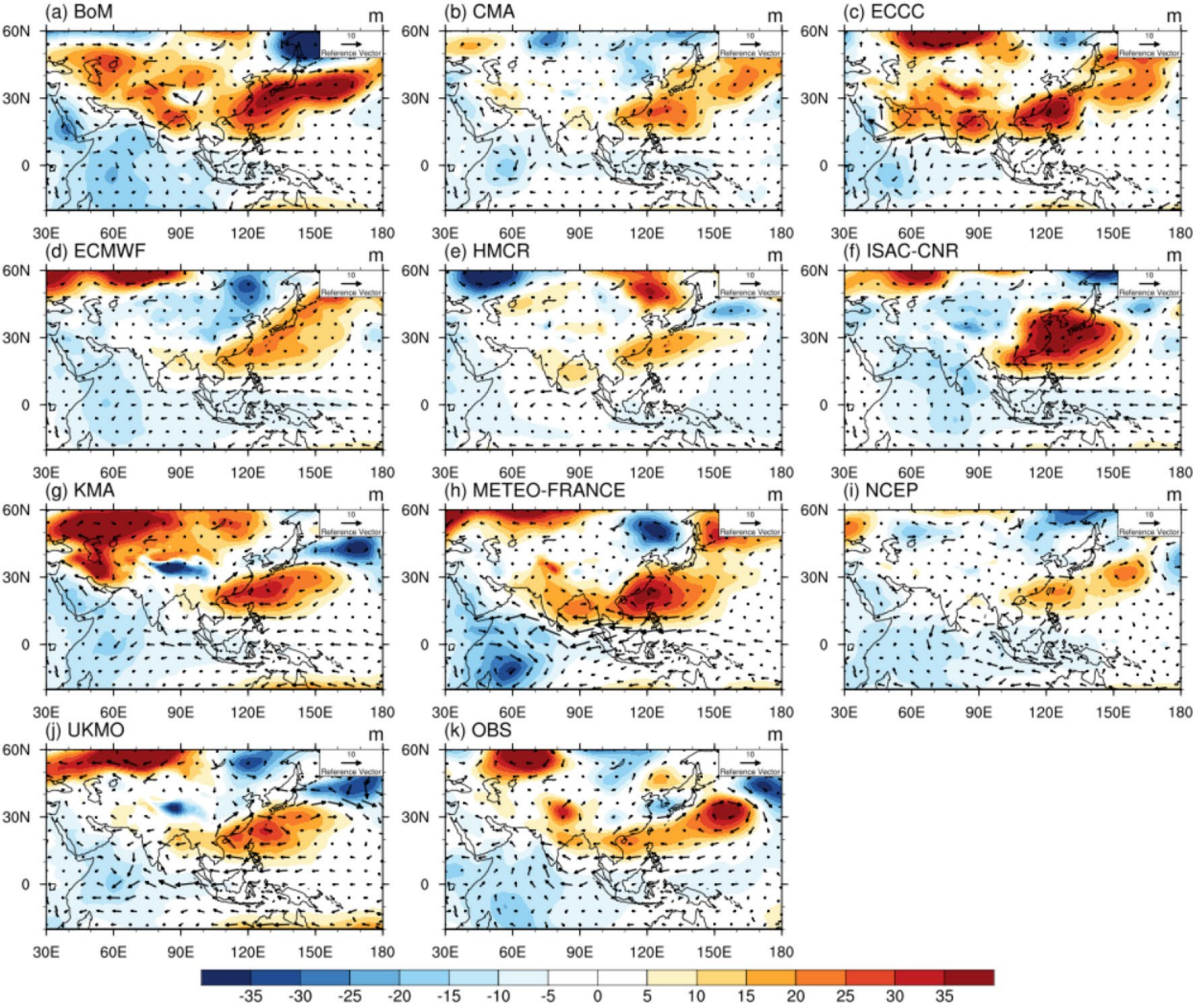
Distribution of 850-hPa geopotential height (shaded, m) and horizontal wind (vector, m s^−1^) anomalies averaged during the forecast period of July 3–25 for the initialization date July 2 in (**a**) BoM, (**b**) CMA, (**c**) ECCC, (**d**) ECMWF, (**e**) HMCR, (**f**) ISAC-CNR, (**g**) KMA, (**h**) METEO-FRANCE, (**i**) NCEP, (**j**) UKMO, and (**k**) observations.

The S2S models with good precipitation forecast performance had well-developed precipitation bands over East Asia, while the poor models did not have a well-defined band shape or had very small precipitation amounts. These differences may be due to the variations in the reproduction of the WPSH. To analyze the characteristics of the WPSH that are important for the formation and location of the precipitation band, the 850-hPa geopotential height and wind field anomaly mean distribution on the initialization date July 2 are shown in Fig. 4. In the observations in Fig. 4k, the WPSH is located in the longitude region 100–160°E, with an east–west extension. The northern edge of the high pressure also appears as an elongated line over East Asia. In addition, the northern edge of the high pressure is clearly visible owing to the low pressure over the Korean Peninsula. The western boundary of the high pressure is located in the South China Sea, which feeds warm and moist air to the precipitation band. KMA and UKMO have western and northern boundaries of the high that are similar to the observations. HMCR and NCEP exhibited the same location but lower intensity. ECCC showed the high pressure splitting in two, but was more robust near the South China Sea. ISAC-CNR and METEO-FRANCE, which exhibited a weak precipitation band, had high pressure over East Asia. BoM and ECMWF, whose precipitation bands were located north of the Korean Peninsula, had WPSH located much further north than observed. Even for the initialization date June 25, the location of the high in ECCC, METEO-FRANCE, and UKMO, where the precipitation band was well defined, was similar to that of the observations, while that in ECMWF, HMCR, and ISAC-CNR, which were among the models with very low precipitation showed the weaker high-pressure anomalies (Supplementary Fig. S3). The BoM, CMA, and KMA exhibited high pressure significantly north of the observations, particularly the BoM and KMA, with East Asia being affected by high pressure.

**Figure 5:**
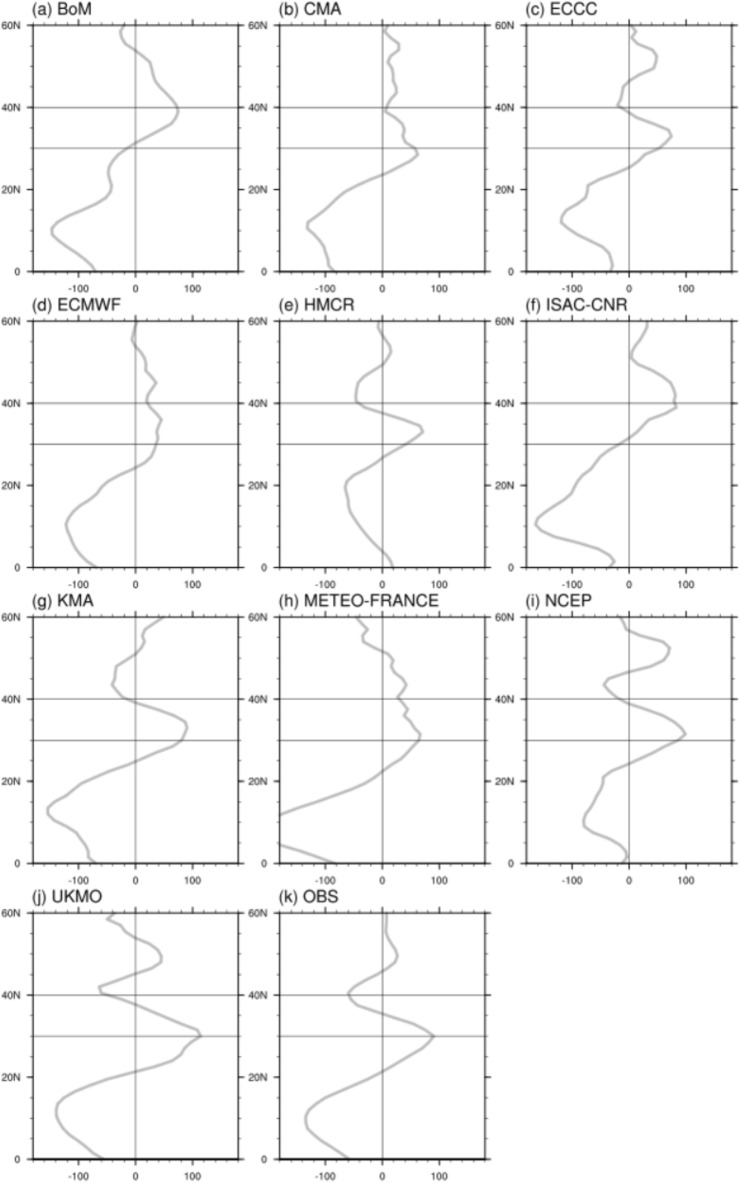
Latitudinal distribution of east–west winds summed over 110–145°E for the initialization date July 2 in (**a**) BoM, (**b**) CMA, (**c**) ECCC, (**d**) ECMWF, (**e**) HMCR, (**f**) ISAC-CNR, (**g**) KMA, (**h**) METEO-FRANCE, (**i**) NCEP, (**j**) UKMO, and (**k**) observations.

To analyze the location and strength of the high pressure in the model, we performed a zonal wind distribution analysis according to latitude, summing the winds from east to west 110–145°E for the initialization date July 2 (Fig. 5). Figure 5k shows the latitudinal zonal wind distribution of the observations, which is zero near latitude 20°N, indicating that this is the central location of the WPSH. Minimum and maximum values of zonal winds were identified at latitudes around 10°N and 30°N, where the wind speeds were maximized to the south and north of the WPSH, respectively. At approximately 35°N, the value of zonal wind was zero owing to the emergence of a low pressure over the Korean Peninsula (Fig. 4k). Precipitation therefore appeared as a distinct line within the 30°N–35°N latitude (Fig. 3k). The UKMO exhibited the southern boundary and center of the WPSH, the northern boundary, and the structure of the cyclone emergence in almost the same location as the observations. The KMAs also exhibited a similar shape, although their positions were slightly northward. The models can therefore be classified into two groups based on center location: below and above 30°N latitude. Examples of models with centers above 30°N include the BoM and ISAC-CNR, whose high pressure extended over the WPSH as well as East Asia (Fig. 5a,f), which is very different from the low-pressure structure over the WPSH and East Asia at lower latitudes that emerged in 2020. We excluded these cases from our analysis. In addition, in certain models where the center of the WPSH emerges at latitudes below 30°N, the point where the wind speed goes back to zero appears at latitudes above 40°N. In these cases, precipitation appears farther north than the observations. For the ECMWF, the transition from positive to negative anticyclonic winds is near latitude 55°N (Fig. 5d), where the northern edge of the high pressure passes over East Asia (Fig. 4d) and the precipitation band is located further north than the study area (Fig. 3d).

In this study, we categorized the models into three groups (Fig. 5 and Supplementary Fig. S4). Models with the center of the WPSH located at latitudes above 30°N were categorized as Group 3. Models at low latitudes where the transition from the northern boundary of the WPSH to the East Asian cyclone occurs at 30–40°N were categorized as Group 1, and as Group 2 if this transition occurs above 40°N. The grouping of the models both the results for the initialization date July 2 and June 25, is presented in Table 1. The pressure structure of the WPSH and the East Asian trough in Group 1 exhibited the highest similarity to the observations. The WPSH was slightly northward in Group 2, and the East Asian region was dominated by the high influence in Group 3. Group 3 was excluded from the statistical analysis because it was completely outside the WPSH forecast for July 2020.

**Table 1:** Clustering S2S models according to WPSH and East Asian precipitation.

Group	Explanation (location)		Initial date	
	Center of WPSH	Transition of WPSH to EA Low	June 25	July 2
1	30°N >	30–40°N	HMCR and UKMO	ECCC, HMCR, KMA, NCEP, and UKMO
2		40°N <	BoM, CMA, ECCC, ISAC-CNR, and METEO-FRANCE	CMA, ECMWF, and METEO-FRANCE
3	30°N <		ECMWF, KMA, and NCEP	BoM and ISAC-CNR

Supplementary Figs. S5 and S6 show the same analysis as that of Fig. 5 using reforecast data for the two initialization dates from 2005 to 2010. These results can be considered as a generalization of the model’s ability to reproduce the WPSH. The reforecast analysis period from 2005 to 2010 did not include years with a strong East Asian monsoon and a developed WPSH. Thus, the observations show that the intensity of the WPSH is weaker than that in the case of 2020. The center of the WPSH was located at a lower latitude than 30°N, and the transition from high to low pressure occurred between 30°N and 40°N, which is the same as in Group 1. The WPSH characteristics of the models were investigated and are summarized in Supplementary Table S1. Group 3 included the models. which were difficult to be included in Groups 1 and 2. For the initial date of June 25, CMA, ECCC, and METEO-FRANCE were classified in Group 2, and for the initial date of July 2, ECCC and UKMO were classified in Group 1 and METEO-FRANCE in Group 2 as in 2020. In addition, in 2020, more models were included in Group 1. The longer number of days affected by the MJO Phases 1–3 are associated with a southerly shift in the center of the WPSH and the location of the northern edge of the WPSH over East Asia.

**Figure 6:**
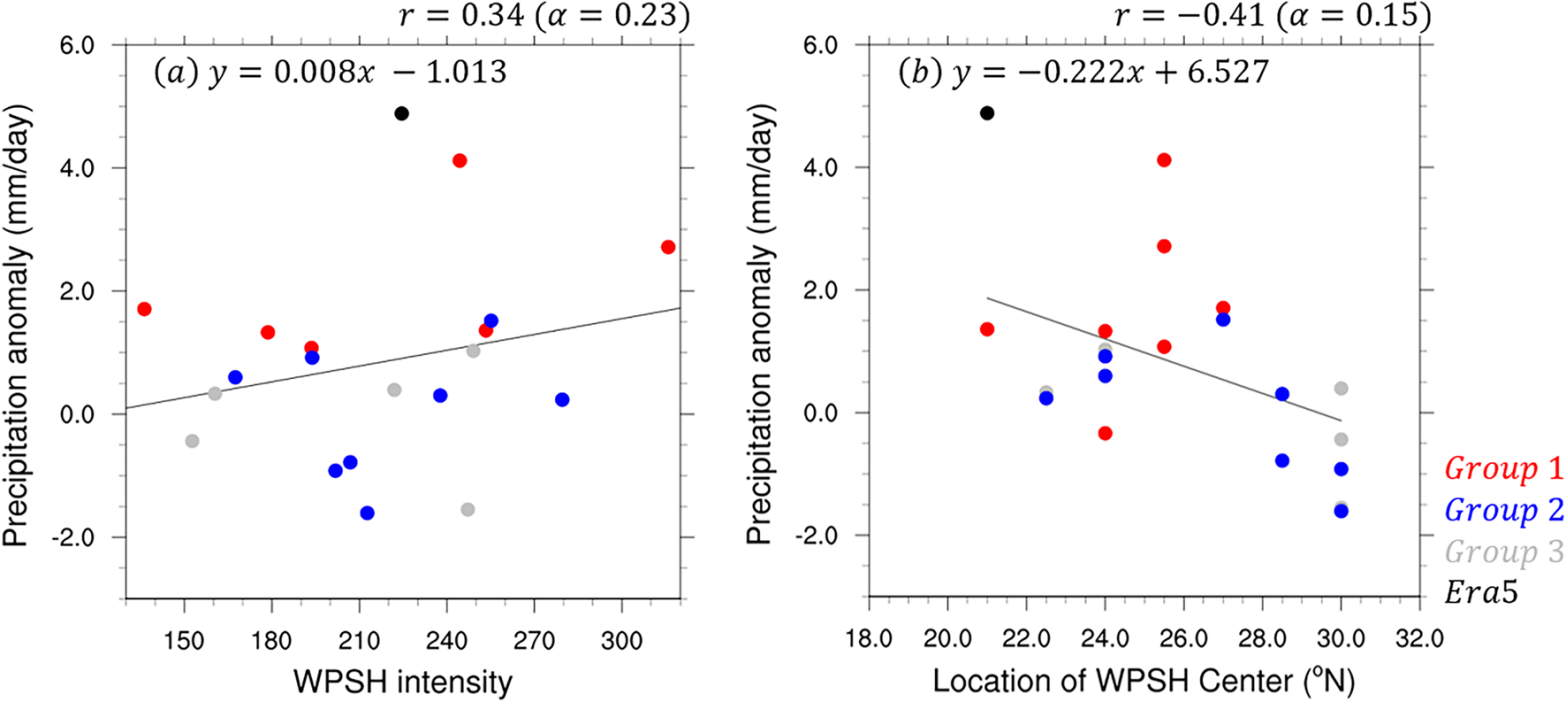
Scatter plot and regression lines between (**a**) WPSH intensity and precipitation (mm day^−1^) and between (**b**) location of the high center and precipitation (mm day^−1^) averaged for the East Asian region over 30–40°N, 110–145°E on initialization dates June 25 and July 2. The red, blue, gray, and black dots indicate Groups 1–3 and observations, respectively. All regression lines, except those for Group 3 and the observations, were analyzed.

The strength of the WPSH is defined as the difference between the maximum and minimum of the zonal wind at 5–45°N latitude as shown by the graphs in Fig. 5 and Supplementary Fig. S3. Because the minimum value of the WPSH is negative, the difference between the maximum and minimum values is equal to the sum of the zonal wind speeds at the southern and northern boundaries of the WPSH. The location of the WPSH center is defined at the first zero latitude value between 20°N and 35°N. To explore the relationship between the characteristics of the WPSH averaged over the analysis period and the performance of precipitation forecasting in East Asia, a scatterplot analysis was performed (Fig. 6a). The WPSH intensity was determined to be approximately 225 m s^−1^ with a precipitation value of approximately 5 mm day^−1^. Groups 1–3 are highlighted in red, blue, and gray, respectively, and the regression line is only analyzed for groups 1 and 2. The correlation coefficient between WPSH intensity and precipitation in East Asia for Groups 1 and 2 is 0.34 (α = 0.23). Group 1 yielded the highest precipitation.

In Fig. 6b, the observations plotted in black show that the center of the WPSH is located at approximately 21°N latitude. Group 1 is located in the upper left corner because its center is located at a low latitude compared to the other groups and exhibits relatively high East Asian precipitation. Group 2 yields less precipitation than Group 1 and the center of the WPSH is also located at relatively high latitudes. Their distributions are negatively correlated, with a correlation coefficient of − 0.41 (α = 0.15). The central location of the WPSH is associated with increased precipitation in East Asia relative to the strength of the WPSH. Therefore, the predictive performance of the S2S model is relatively dependent on the strength and location of the center of the WPSH reproduced by the model. Although the two correlation coefficients shown in Fig. 6 are not statistically significant, the relationship between WPSH and East Asian precipitation based on different models and observations provides the necessary information for improving model performance.

### Role of Madden–Julian Oscillation in precipitation prediction

To determine the role of the MJO in East Asian precipitation, we first performed a phase analysis of the MJO during the analysis period (Supplementary Figs. S7 and S8). The KMA model was excluded from the analysis because it does not provide OLR data. The observations show that the MJO remained in phases 1–4 during the analysis period, with most of the periods in an active state and at an amplitude of > 1 (Supplementary Fig. S7j). The UKMO, which has very similar WPSH characteristics to the observations, mainly remained in phases 1–3, but became inactive in late July. Most of the S2S models were mostly in Phase 2 at the beginning of the analysis period, but moved to other phases or became inactive. The initialization date June 25 was characterized by a significant decrease in the MJO amplitude of the models (Supplementary Fig. S8), and these models did not have a similar MJO phase as that on the initialization date July 2. Most S2S models struggled to predict the long-duration MJO over the Indian Ocean, leading to weakened WHSP and East Asian rainband.

**Figure 7:**
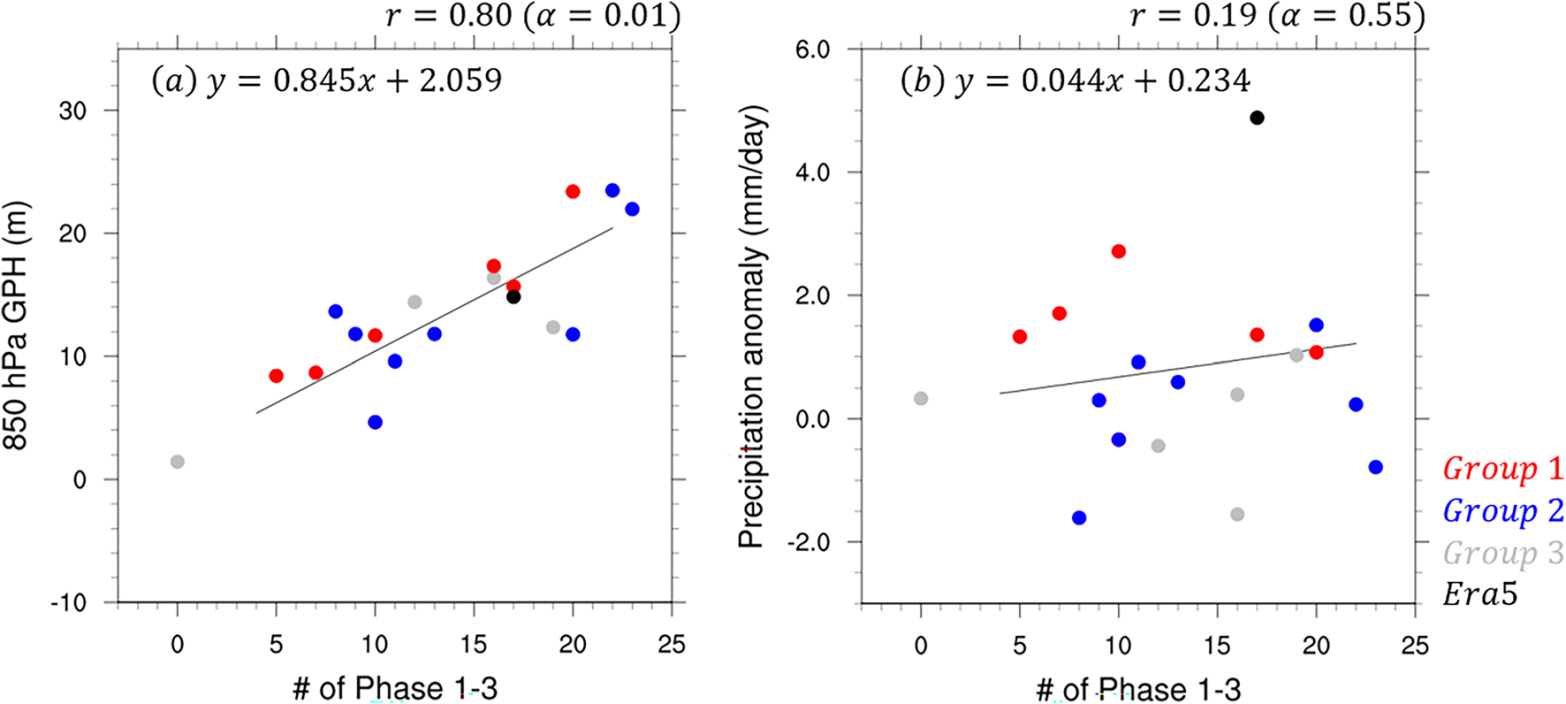
Scatter plot and regression lines between (**a**) geopotential height averaged for the western part of WPSH and number of MJO Phases 1–3 active days and between (**b**) precipitation (mm day^−1^) averaged for the East Asian region over 30–40°N, 110–145°E, and number of MJO Phases 1–3 active days on the initialization dates June 25 and July 2. The red, blue, gray, and black dots indicate Groups 1–3 and the observations, respectively. All regression lines, except those for Group 3 and the observations, were analyzed.

When MJO Phases 1–3 are activated, the WPSH is reinforced and East Asian precipitation increases^10–13^. One way of identifying the reinforcement of the WPSH is by the western extension of the WPSH. A scatter plot analysis of the 850-hPa geopotential height over the 15°N–25°N, 90°E–120°E region averaged over the analysis period and MJO Phases 1–3 active days showed a very high positive correlation between these variables, with a correlation coefficient of 0.80 (α = 0.01) (Fig. 7a). For Group 1, MJO Phases 1–3 had fewer active days and were less extended west of the WPSH. In addition, more active days of MJO Phases 1–3 were associated with increased precipitation, with a correlation coefficient of 0.19 (α = 0.55) (Fig. 7b). Group 1 exhibited more precipitation despite having fewer active days, while Group 2 exhibited less precipitation despite having more active days. This suggests that while the western extension of the WPSH associated with MJO Phases 1–3 active days is well simulated, the East Asian precipitation associated with the MJO was highly model dependent. because it is not the only factor influencing variability in East Asian precipitation, but rather it indirectly impacts through the WPSH. Models that adequately simulate the location of the WPSH, such as Group 1, suggest that their performance in predicting East Asian precipitation is improving despite few MJO active days. If these models reproduce the number of MJO activity days closer to reality, the WPSH will strengthen and contribute to increased precipitation in East Asia.

To examine the model’s WPSH and East Asian precipitation forecast characteristics for MJO Phases 1–3 active days of the S2S model, a composite analysis was performed. Supplementary Figs. S9 and S10 show the results for the initialization dates July 2 and June 25, respectively. Generally, the precipitation bands were pronounced compared to the previous analysis period. This characteristic is most evident in the NCEP model (Group 1) with the initialization date July 2 (Fig. S9h). This model has very small MJO Phases 1–3 active days, in the order of 5 days (Fig. S7h), which results in a very blurred precipitation band upon averaging (Fig. 3i). The METEO-FRANCE model (Group 2) with the initialization date July 2 had no distinct precipitation band, and no significant difference between the mean of the analysis period or mean of the MJO active days. Compared to the other models, this model has numerous MJO Phases 1–3 active days, and enormous amplitude (Supplementary Fig. S9g).

Examination of the high pressure and wind distributions on the active days of MJO Phases 1–3 revealed an intensification of the WPSH (Supplementary Figs. S11 and S12). As the western boundary of the WPSH in the observations extended westward into the South China Sea, the high pressure intensified in an east–west direction in Fig. 4, and the southwesterly winds that reached the East Asian region originated in Southeast Asia (Fig. S11j). WPSH intensity and the accompanying anti-cyclonic circulation in the models were reinforced. The NCEP model on the initialization date July 2 mentioned in the precipitation composite analysis shows a very pronounced WPSH (Fig. 4i and Supplementary Fig. S11h). The underestimated precipitation in the NCEP model is associated with a very weak WPSH owing to the unusually low number of days of active MJO days in phases 1–3. METEO-FRANCE also showed very strong development of WPSH intensity. In this model, the reinforcement of the WPSH caused the high pressure to extend in a north–south direction and a large area was affected by the high pressure.

**Figure 8:**
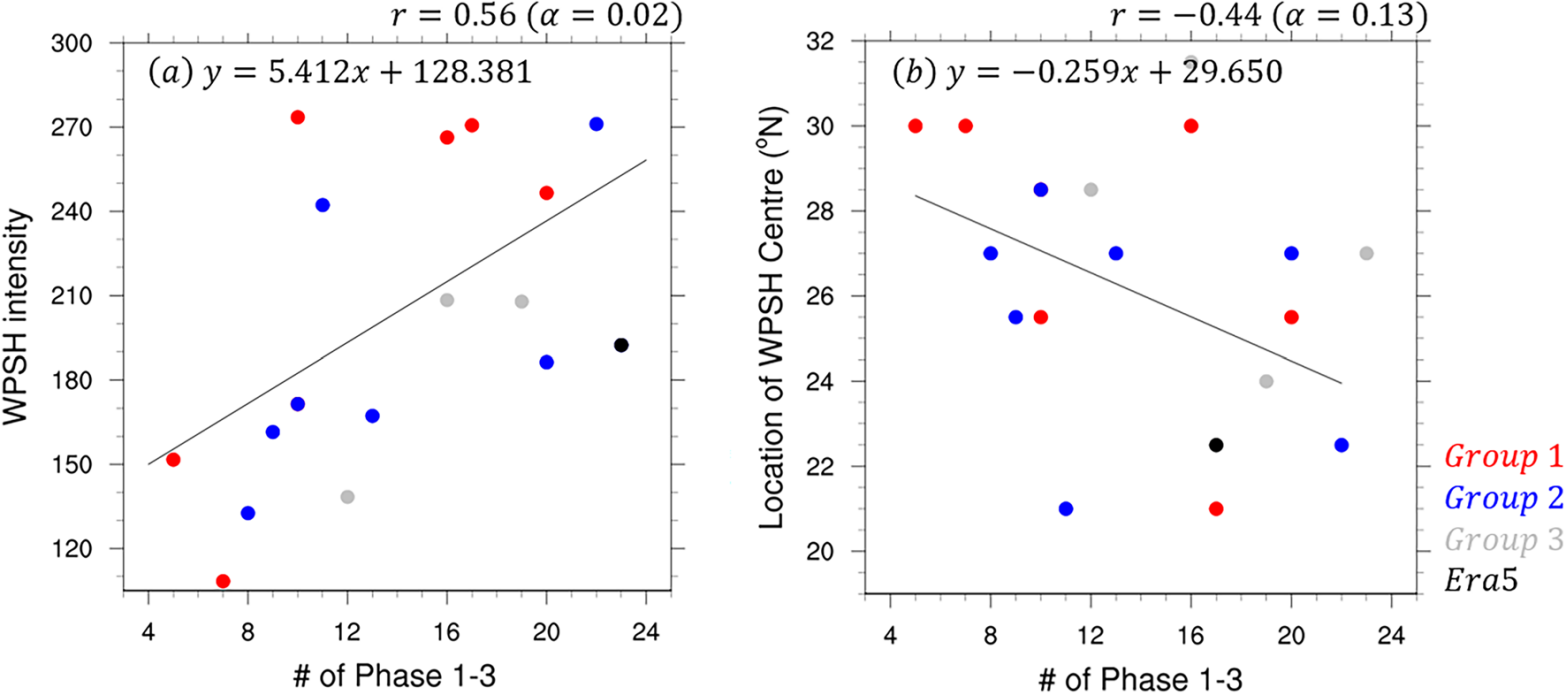
Scatter plot and regression line between (**a**) WPSH intensity and number of active days MJO Phases 1–3 and between (**b**) location of the high center and number of active days of MJO Phases 1–3 at the initialization dates June 25 and July 2. The red, blue, gray, and black dots indicate Groups 1–3 and observations, respectively. All regression lines, except those for Group 3 and the observations were analyzed.

We performed a scatter plot analysis of the high-pressure characteristics of the WPSH on the active days of MJO Phases 1–3 (Fig. 8). The intensity of the WPSH tended to increase as the number of MJO Phases 1–3 active days increased (correlation coefficient = 0.56, α = 0.02), which was associated with the westward expansion of the WPSH during the analysis period (Fig. 8a). In Group 1, the location of the WPSH was similar to that of the observations made; however, the pressure was very low in several models. However, the realistic simulation of the number of MJO days would contribute to increased precipitation owing to the reinforcement of the high pressure. As the number of MJO active days increases, the center of the WPSH reaches lower latitudes (correlation coefficient = -0.44, α = 0.13). In Group 1, the center of the WPSH for several models was at a fairly high latitude (Fig. 8b) and increasing the number of active days of MJO Phases 1–3 reinforces the WPSH and moves its center southward. Therefore, increasing the number of MJO active days would realistically simulate the WPSH in S2S models, which would help improve the performance of summer precipitation forecasts over East Asia in July 2020.

To characterize the WPSH forecast, we also conducted a root-mean-square-error (RMSE) analysis of the intensity and center position of WPSH against the observations from June 1 to July 31, 2020 (Supplementary Fig. S13). Here, we applied a moving average from prediction day n to n + 13 days to remove the short-term fluctuations of daily anomalies. The accuracy of predicting the intensity of the WPSH decreases as the forecast time increases for all S2S models. However, the errors for the WPSH center are quite large even at the beginning of the forecast, which indicates particular difficulties in forecasting for the position of the WPSH. The models with lowest errors are ISAC-CNR, KMA, and ECMWF for WPSH intensity and BoM, ECCC, ISAC-CNR, and METEO-FRANCE for WPSH center. Compared to KMA and UKMO, which have good forecast performance for the East Asian summer monsoon, it is critical to reproduce the teleconnection of the MJO with the monsoon system to predict the anomalous rainfall in East Asia.

## Conclusion

In this study, the role of MJOs in precipitation forecasting in East Asia was analyzed using S2S models. East Asian precipitation intensity was positively correlated with WPSH intensity and negatively correlated with the location of the center of the WPSH. WPSH is reinforced by inducing downdrafts in the WPSH during MJO Phases 1–3, and the S2S model showed a positive correlation between the number of MJO active days and the westward extension of the WPSH during the analysis period. As the number of MJO active day increases, the WPSH intensity increases and the center of the WPSH moves toward lower latitudes. S2S models need to simulate the MJO more realistically to ensure that the WPSH intensity is high and the center remains at low latitudes, such as in July 2020.

In the present study, the significance of a realistic representation of the MJO for the enhancement of East Asian precipitation in 2020 was demonstrated. However, attributing the improvement in East Asian precipitation forecasting performance to the simulation of the MJO alone would be erroneous. The development of the WPSH and the location of the northern boundary of the WPSH may also have been influenced by the presence of the East Asian low. This study did not address whether the northerly position of the center of the WPSH and the position of the precipitation band in Group 2 of the S2S models was due to differences in the representation of the MJO or a model property, and this warrants further investigation. Furthermore, further experiments on the deep-convection development of the MJO and the accompanying warm sea surface temperatures are needed to understand how certain phases of the MJO emerge in the long term. Notably, the Group 1 models with weaker WPSHs may have been influenced by characteristics of the region in the vicinity of the WPSH, such as SSTs or convection in the western Pacific. Investigating the role of mid-to-high latitude forcing in the 2020 rainband in the future is necessary, as the East Asian rainband is influenced not only by tropical factors but also by mid-to-high latitude forcing^40^. Nonetheless, this study analyzes the performance of S2S models over East Asian precipitation in July 2020 and its association with the characteristics of WPSH development on the MJO active days.

Finally, S2S models can enhance the forecast performance of the East Asian summer monsoon by accurately reproducing the strength and location of the WPSH. It is also crucial to realistically simulate the strength and phase of the MJO, as there are linkages between the MJO, the WPSH, and the East Asian summer monsoon. The MJO is not a unique factor influencing the strength of the WPSH or the East Asian summer monsoon, but the July 2020 event was selected for its record-breaking summer rainfall as well as persistent MJO activity. Therefore, a more comprehensive multi-case analysis is necessary to ensure the robustness of our results. If there are additional cases where the MJO, the WPSH, and the East Asian summer monsoon are closely linked, a statistical analysis can be conducted to investigate their relationship. Furthermore, sensitivity experiments on the teleconnection of the MJO using forecast models can be performed. This is expected to provide more useful information on the characteristics of the performance of S2S models in predicting the teleconnection of MJOs and to improve their predictability.

## Data and methods

The study analyzed data from 10 models utilized in the S2S project: BoM, CMA, ECCC, ECMWF, HMCR, ISAC-CNR, METEO-FRANCE, KMA, NCEP, and UKMO (Table 2). JMA was excluded from the analysis because its forecast time differed from that of other models. With the exception of the BoM model, data from the remaining models are all available at a horizontal resolution of 1.5º × 1.5º; therefore, BoM was regridded to a common grid. We selected two initialization dates to forecast this period: June 25 and July 2. The earlier the initialization date, the longer the lead-time for the analysis period. Here, ECCC and METEO-FRANCE could not cover the whole of July because the forecast period was 32 days for the initialization date June 25. Therefore, the analysis was performed from July 3–25, which is the maximum period that could be analyzed.

**Table 2:** Resolution and analysis periods of the S2S models in this study.

Model	Resolution	Real-time	Reforecast	Forecast period (day)
BoM	T47 L17	June 25 and July 2, 2020	1981–2013	62
CMA	T225 L56		2005–2019	60
ECCC	Yin-Yang grid at 0.35° uniform resolution (~ 39 km) L85		1998–2017	32
ECMWF	Tco639 L137 (approximately 16 km) up to day 15 and Tco319 (approximately 32 km) after day 15		2000–2019	46
HMCR	0.9° × 0.72° L96		1985–2010	60
ISAC-CNR	0.8° × 0.56° L54		1981–2010	32
KMA	N216 L85 (0.83° × 0.56°, approximately 60 km in mid latitudes)		1991–2016	61
METEO-FRANCE	TL255 (approximately 80 km)		1993–2014	32
NCEP	T126 L64 (approximately 100 km)		1999–2010	44
UKMO	N216 L85		1993–2016	60

The model's anomaly is calculated using reforecasts, the duration of which varies according to the model. Therefore, the quantitative comparison of the forecast performance of the models is complicated. Moreover, because this study is not an intercomparison or evaluation of S2S models, but rather an analysis of the relationship between the models' MJO forecast performance and East Asian precipitation, the inconsistency of the reforecast periods does not impede the analysis of the results. For each initialization date and forecast time, anomaly was calculated by averaging available reforecast data for the entire period, and then subtracting each forecast time if the initialization date of the real-time data and the reforecast data matched. If no reforecast data had an initialization date that matched that of the real-time data, the closest previous initialization date was selected.

The model's precipitation data are provided as cumulative precipitation values at 6-h intervals; therefore, we converted them to daily data. The cumulative longwave radiation flux data were converted to daily data, and negative values were regarded as outgoing longwave radiation (OLR). Geopotential height and horizontal wind were also considered in the analysis. For comparison with the model data, ERA5 data corresponding to the analysis period were used^41^, and anomalies were calculated for the period from 1991 to 2020.

The MJO index of the observations was calculated using the method described by Wheeler and Hendon^42^. First, the daily index components of the real-time multivariate MJO (RMM), RMM1, and RMM2 were calculated using ERA5 data. The means and harmonics of OLR, 200-hPa zonal wind (U200), and 850-hPa zonal wind (U850) were calculated for the period 1991–2020, and the values averaged over the previous 120 days were removed from the day targeted for calculation. Because the model forecast periods are short (one to two months), the preceding 120 days were continued from the forecast initialization date, and the 120-day mean value for each forecast day was subtracted to remove variations with periods below 120 days. The 15S–15°N region was then averaged and normalized using the normalized factor from Gottschalk et al.^43^. The RMM1 and RMM2 of the model’s prediction time were obtained by calculating the empirical orthogonal function for the observations and dividing by the standard deviation of the model.

### Supplementary Information

Supplementary Information.

## Data Availability

The S2S model and ERA5 data were provided by the European Centre for Medium-Range Weather Forecasts (ECMWF) at https://apps.ecmwf.int/datasets/data/s2s/ and https://www.ecmwf.int/en/forecasts/dataset/ecmwf-reanalysis-v5, respectively.
