# Supplementary material for: Role of Madden–Julian Oscillation in predicting the 2020 East Asian summer precipitation in subseasonal-to-seasonal models
Source: Sci Rep. 2024 Jan 9;14:865. doi: 10.1038/s41598-024-51506-9 (PMC10776580; doi:10.1038/s41598-024-51506-9)
Supplement: Supplementary file 1 — Supplementary Information. [file 41598_2024_51506_MOESM1_ESM.docx]

**Role of Madden–Julian Oscillation in predicting the 2020 East Asian summer precipitation in subseasonal-to-seasonal models**

**Jieun Wie, Jinhee Kang, Byung-Kwon Moon***

Division of Science Education / Institute of Fusion Science, Jeonbuk National University, Jeonju 54896, South Korea

*Corresponding author: Prof. Byung-Kwon Moon

Email: [moonbk@jbnu.ac.kr](mailto:moonbk@jbnu.ac.kr)

**Supplementary Information**

**Table S1.** Same as Table 1 but of the reforecast data.

| Group | Explanation (location) | | Initial date | |
| --- | --- | --- | --- | --- |
|  | Center of WPSH | Transition of WPSH to EA Low | June 25 | July 2 |
| 1 | 30°N > | 30–40°N | ECMWF | ECCC, UKMO |
| 2 |  | 40°N < | CMA, ECCC, KMA, METEO-FRANCE, UKMO | KMA, METEO-FRANCE, NCEP |
| 3 |  |  | BoM, HMCR, ISAC-CNR, NCEP | BoM, CMA, ECMWF, HMCR, ISAC-CNR |


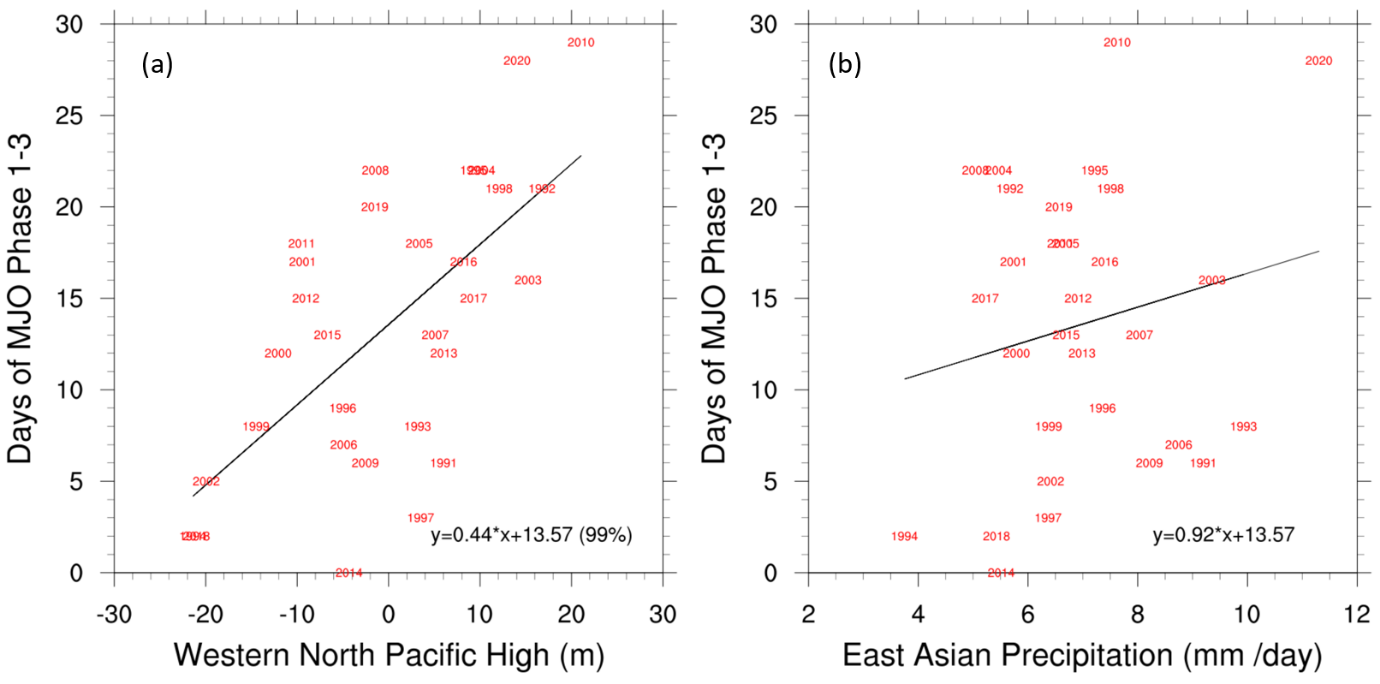


**Figure S1.** Scatter plot and regression lines between (a) WPSH intensity averaged the region over 15–25°N, 105–145°E and number of MJO Phases 1–3 active days and between (b) precipitation (mm day^-1^) averaged East Asian region over 30–40°N, 110–145°E and number of MJO Phases 1–3 active days in ERA5 in July during 1991-2020. The numbers of the figure represent years and the black lines indicate regression lines.


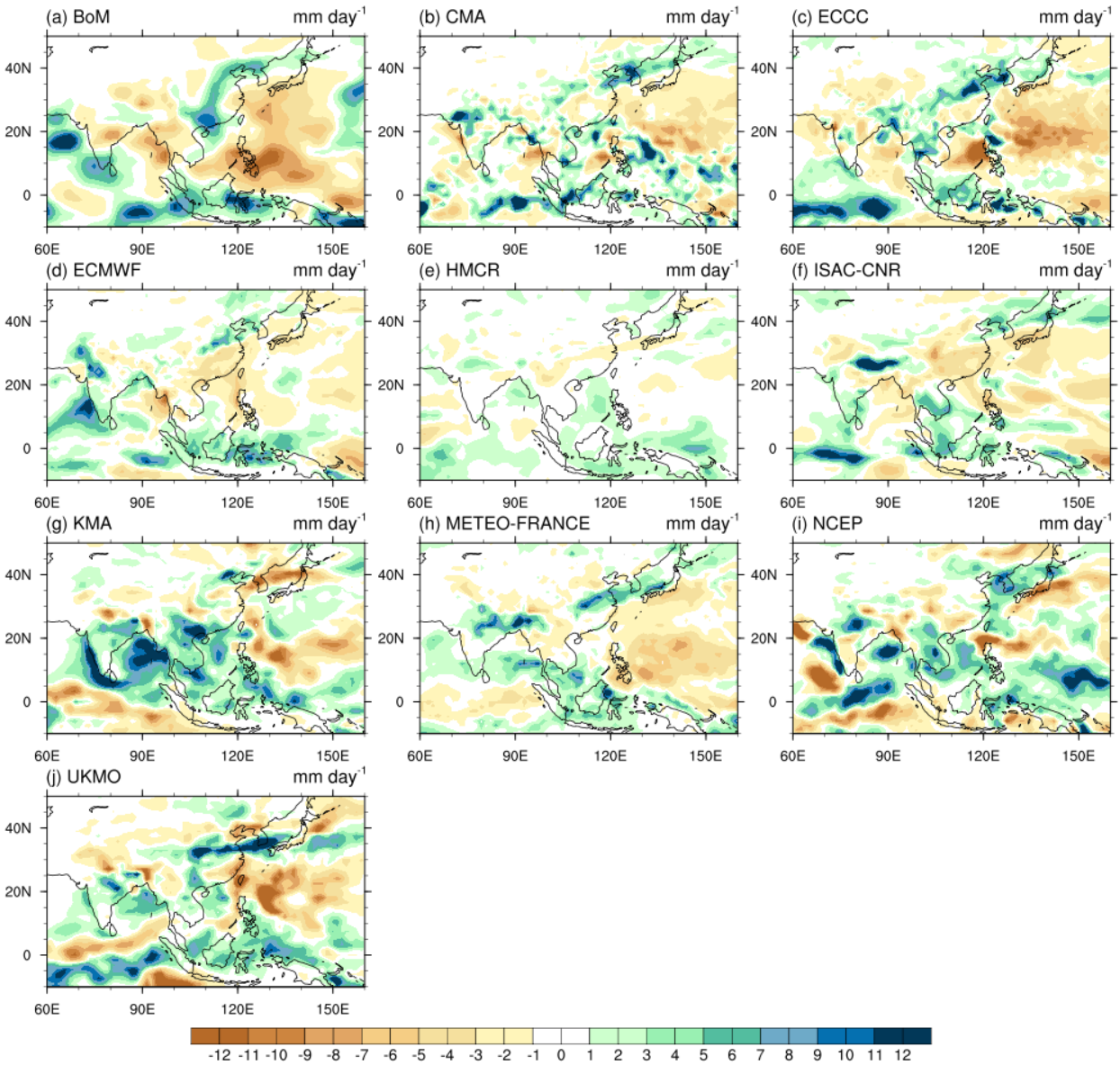


**Figure S2.** Same as Fig. 3 but for the initialization date June 25, 2020.


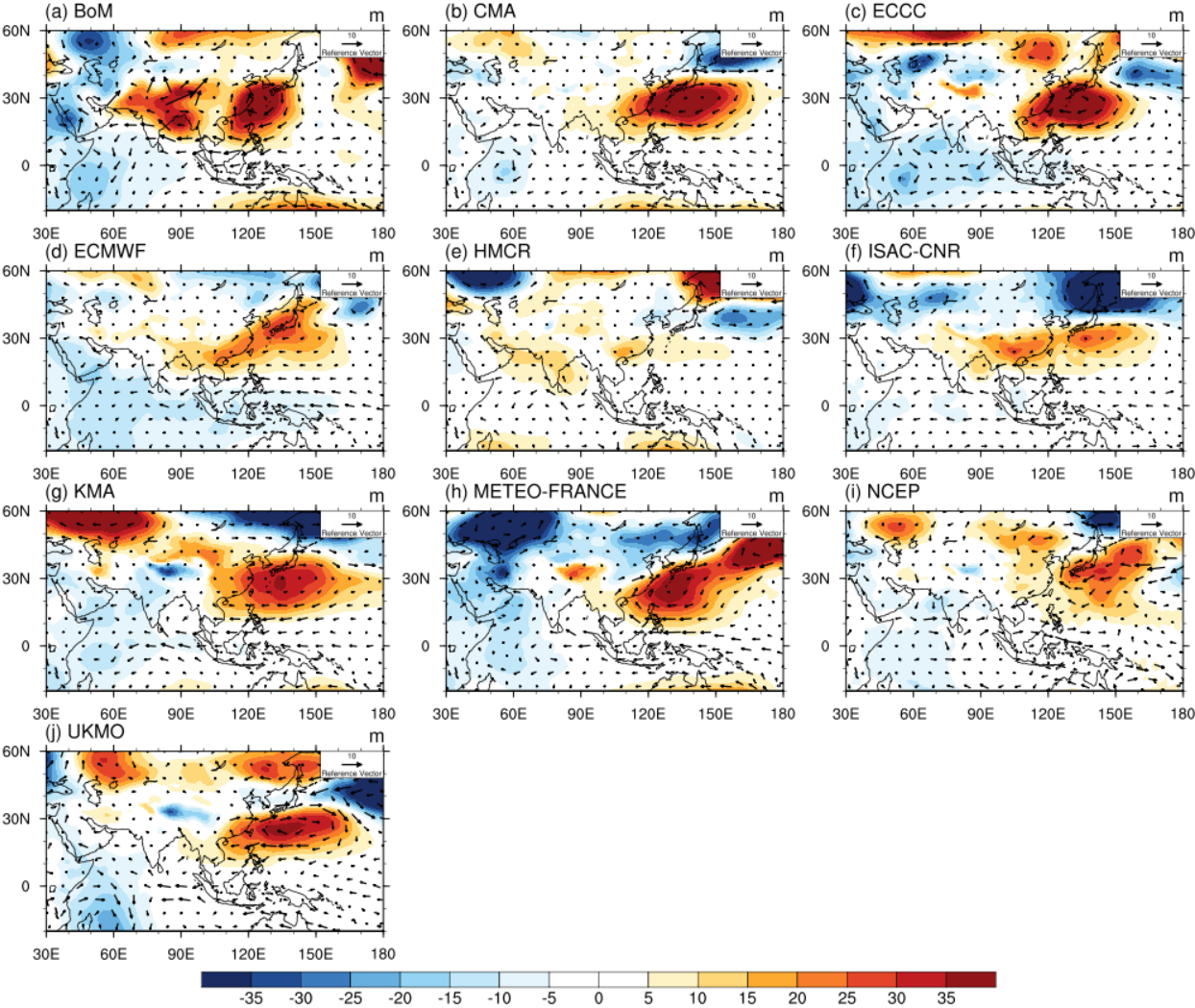


**Figure S3.** Same as Fig. 4 but for the initialization date June 25, 2020.


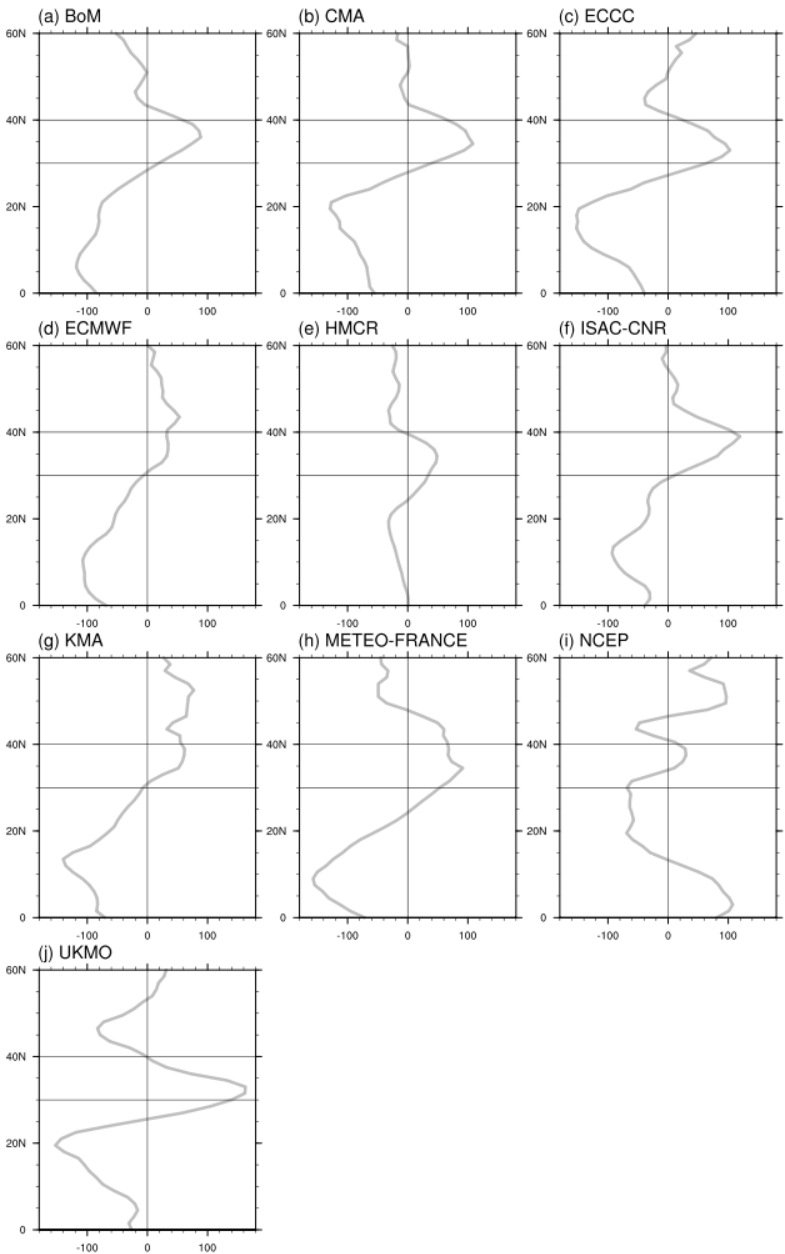


**Figure S4.** Same as Fig. 5 but for the initialization date June 25, 2020.


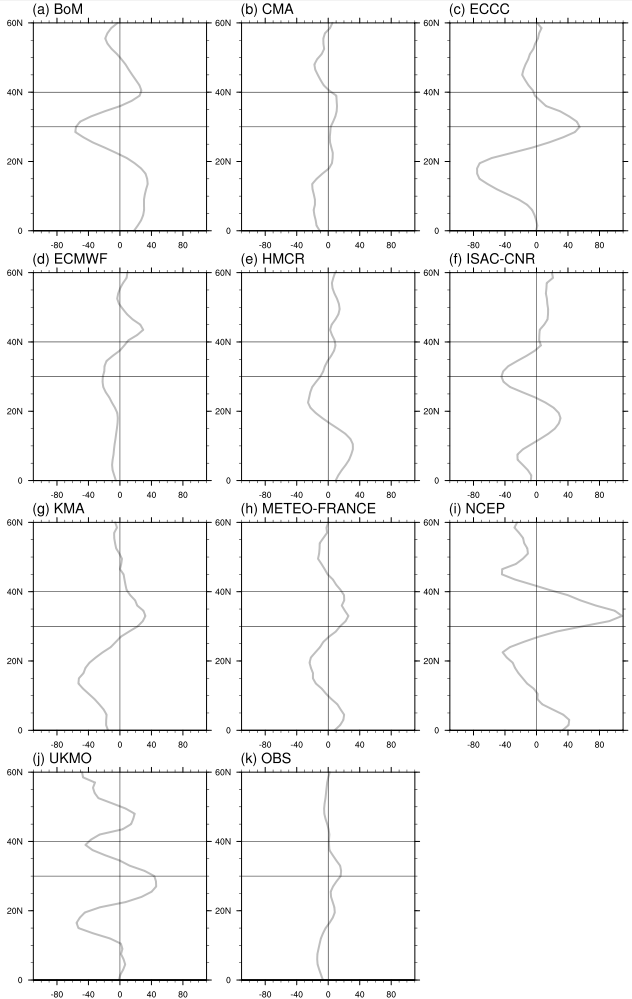


**Figure S5.** Same as Fig. 5 but of reforecast data for the initialization date July 2, 2005-2010.


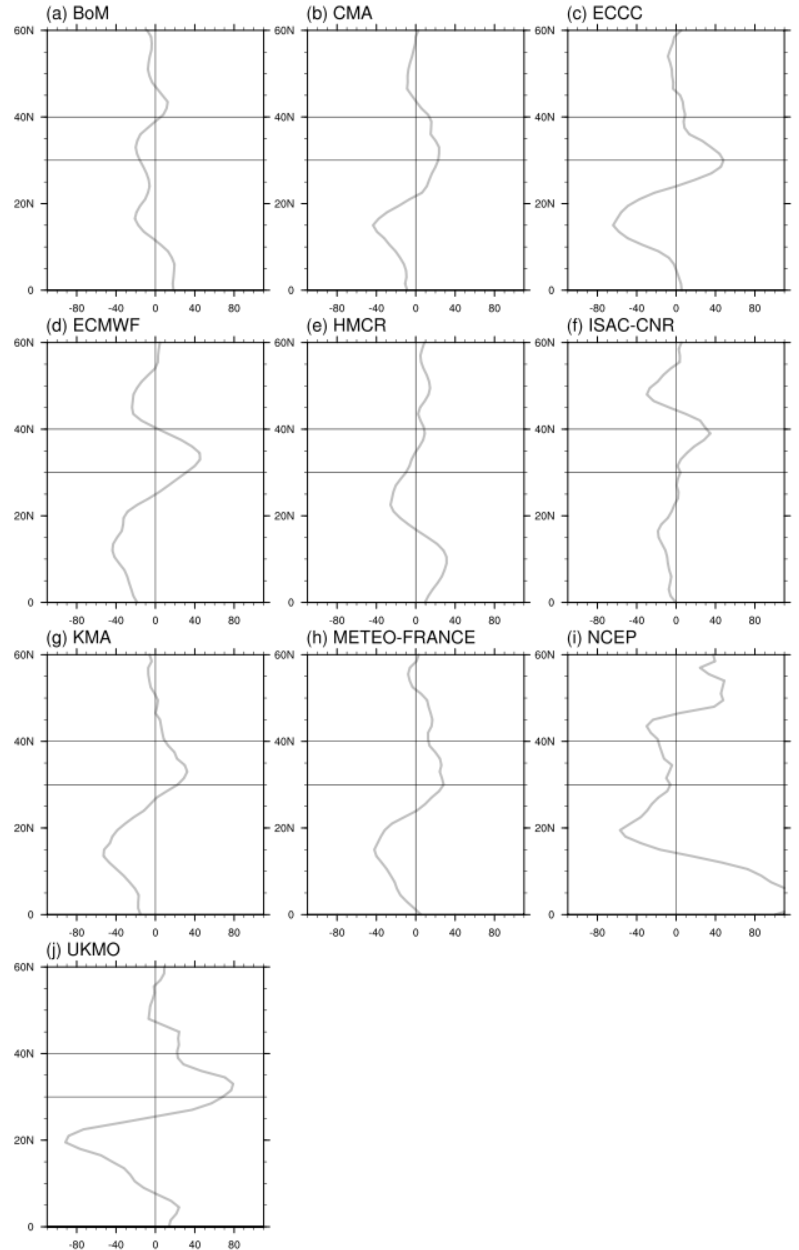


**Figure S6.** Same as Fig. 5 but of reforecast data on the initialization date June 25, 2005-2010.


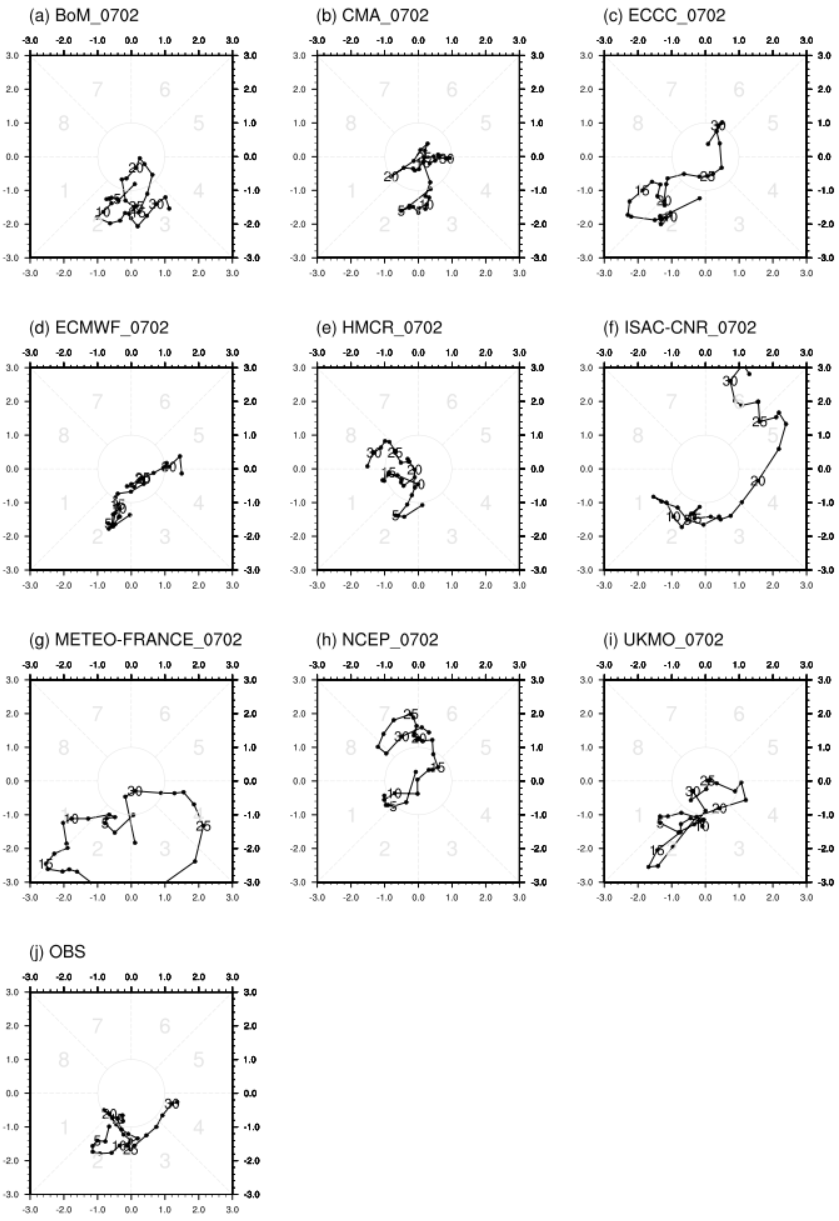


**Figure S7.** Madden–Julian Oscillation (MJO) phase space diagrams in (a) BoM, (b) CMA, (c) ECCC, (d) ECMWF, (e) HMCR, (f) ISAC-CNR, (g) METEO-FRANCE, (h) NCEP, (i) UKMO, and (j) observation on the initialization date July 2, 2020 during forecast times from July 3 to 31, 2020. The numbers on the graph represent dates in July.


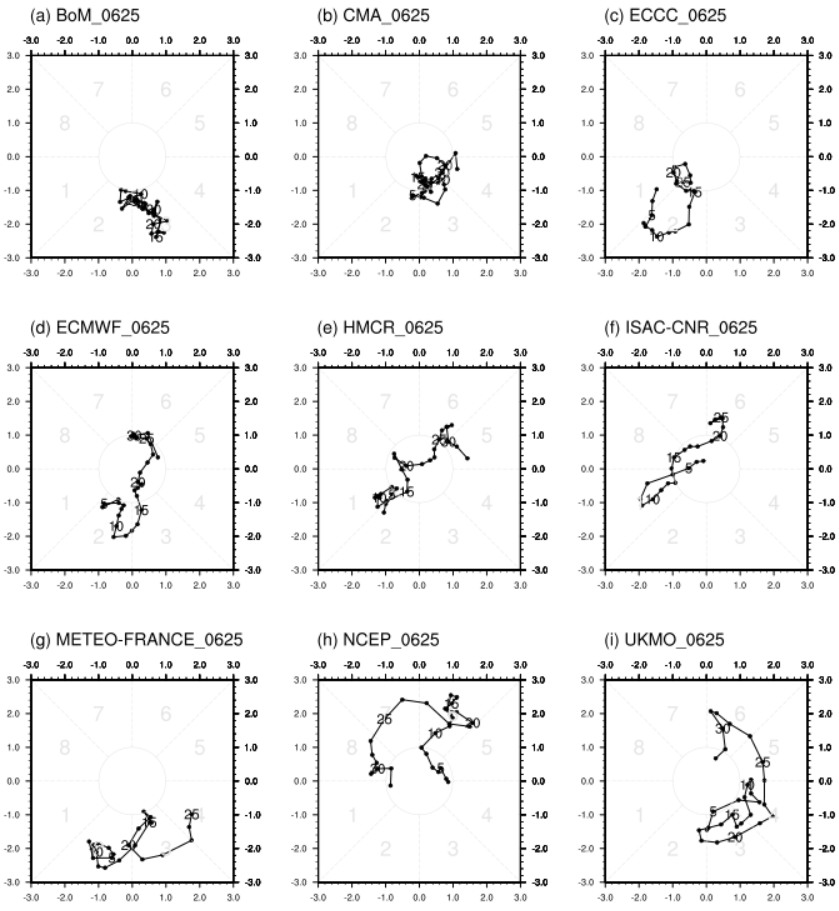


**Figure S8.** Same as Fig. S7 but for the initialization date June 25, 2020.


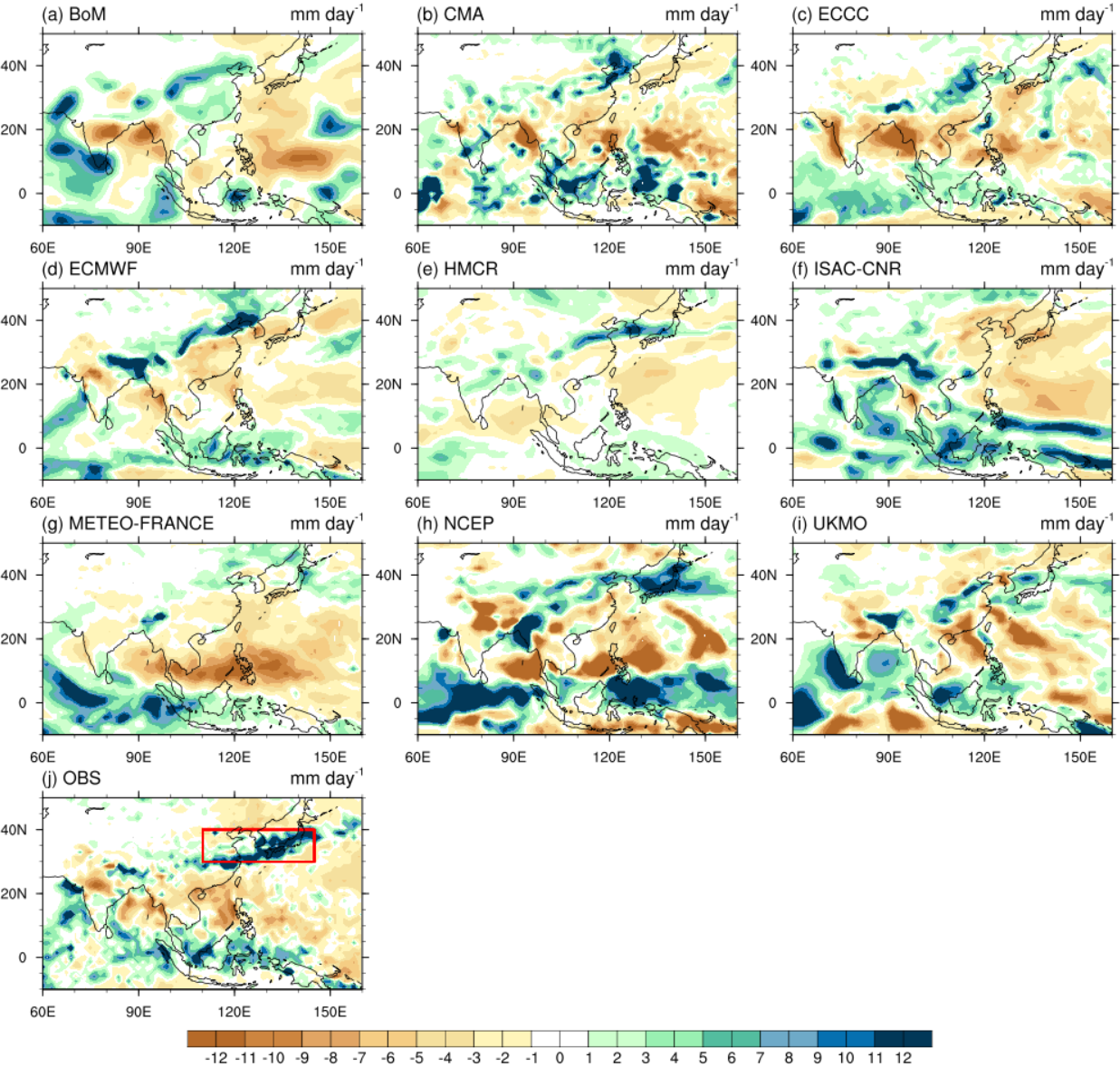


**Figure S9.** Composite precipitation anomalies (mm day^-1^) during MJO Phases 1–3 active days for (a) BoM, (b) CMA, (c) ECCC, (d) ECMWF, (e) HMCR, (f) ISAC-CNR, (g)METEO-FRANCE, (h) NCEP, (i) UKMO, and (j) observation on the initialization date July 2 averaged from July 3 to 25, 2020. The red box in (m) indicates the East Asian region.


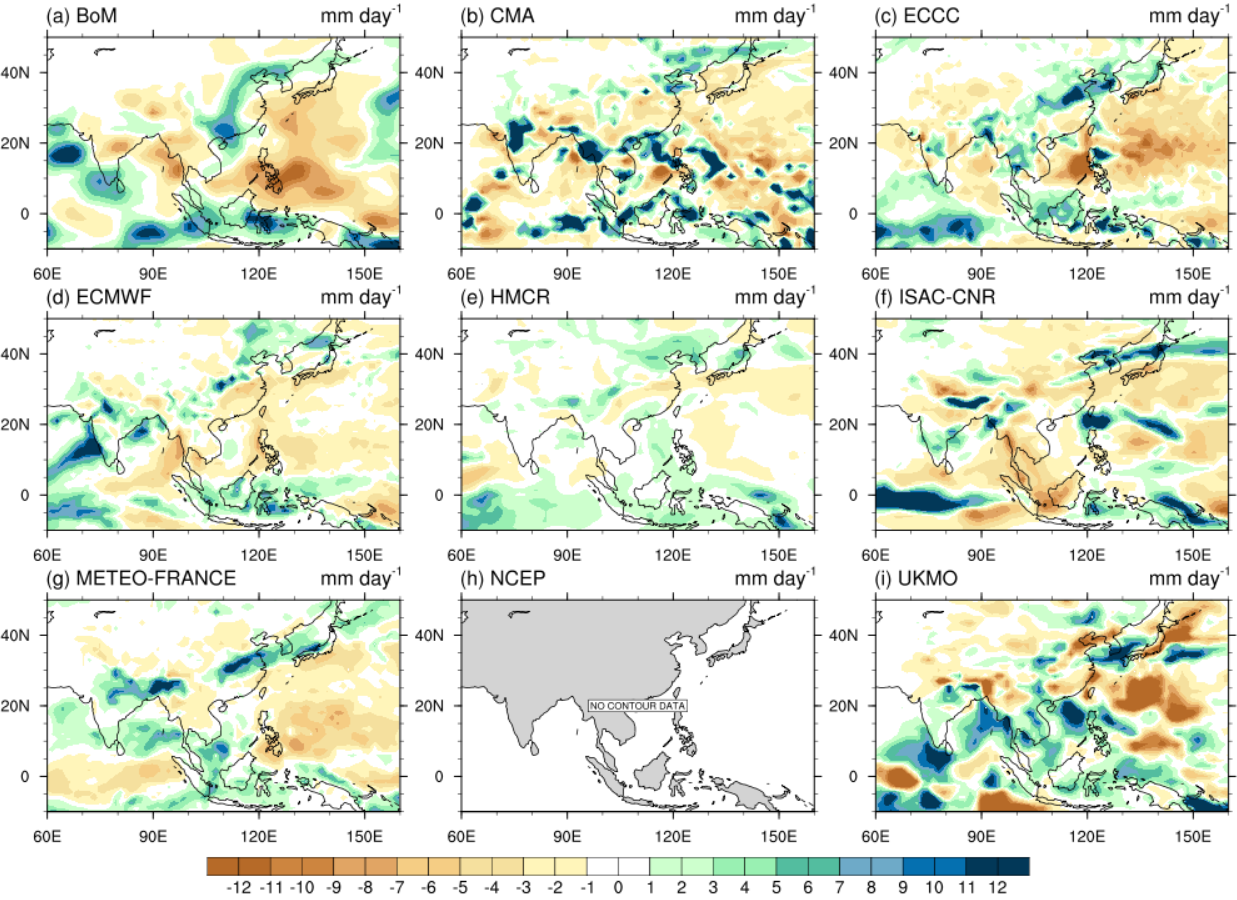


**Figure S10.** Same as Fig. S9, but for the initialization date June 25, 2020. NCEP had no MJO active days.


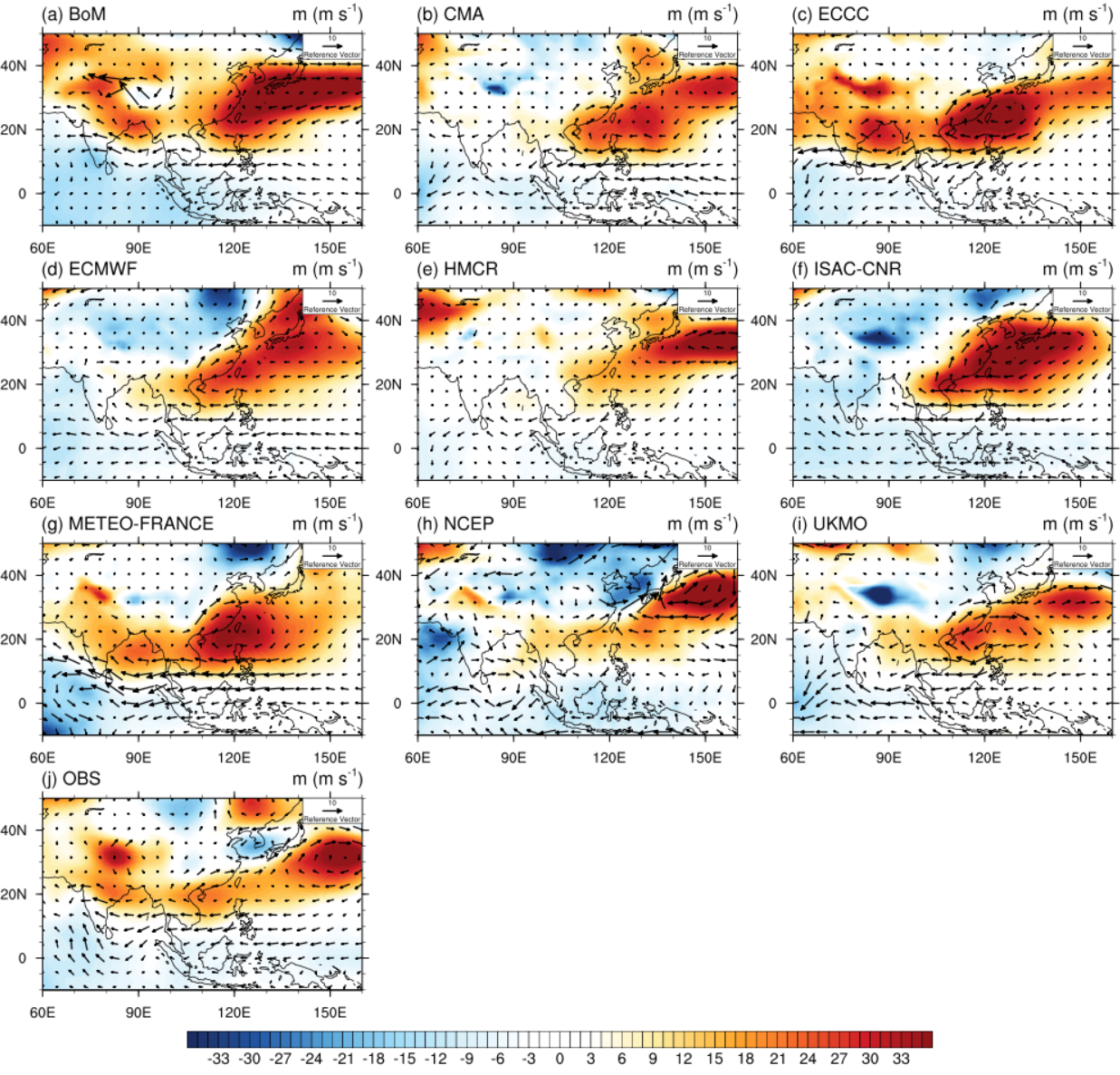


**Figure S11.** Composite 850-hPa geopotential height (shaded, m) and horizontal wind (vector, m s^-1^) anomalies during MJO Phases 1–3 active days for (a) BoM, (b) CMA, (c) ECCC, (d) ECMWF, (e) HMCR, (f) ISAC-CNR, (g)METEO-FRANCE, (h) NCEP, (i) UKMO, and (j) observation on the initialization date July 2 averaged from July 3 to 25, 2020.


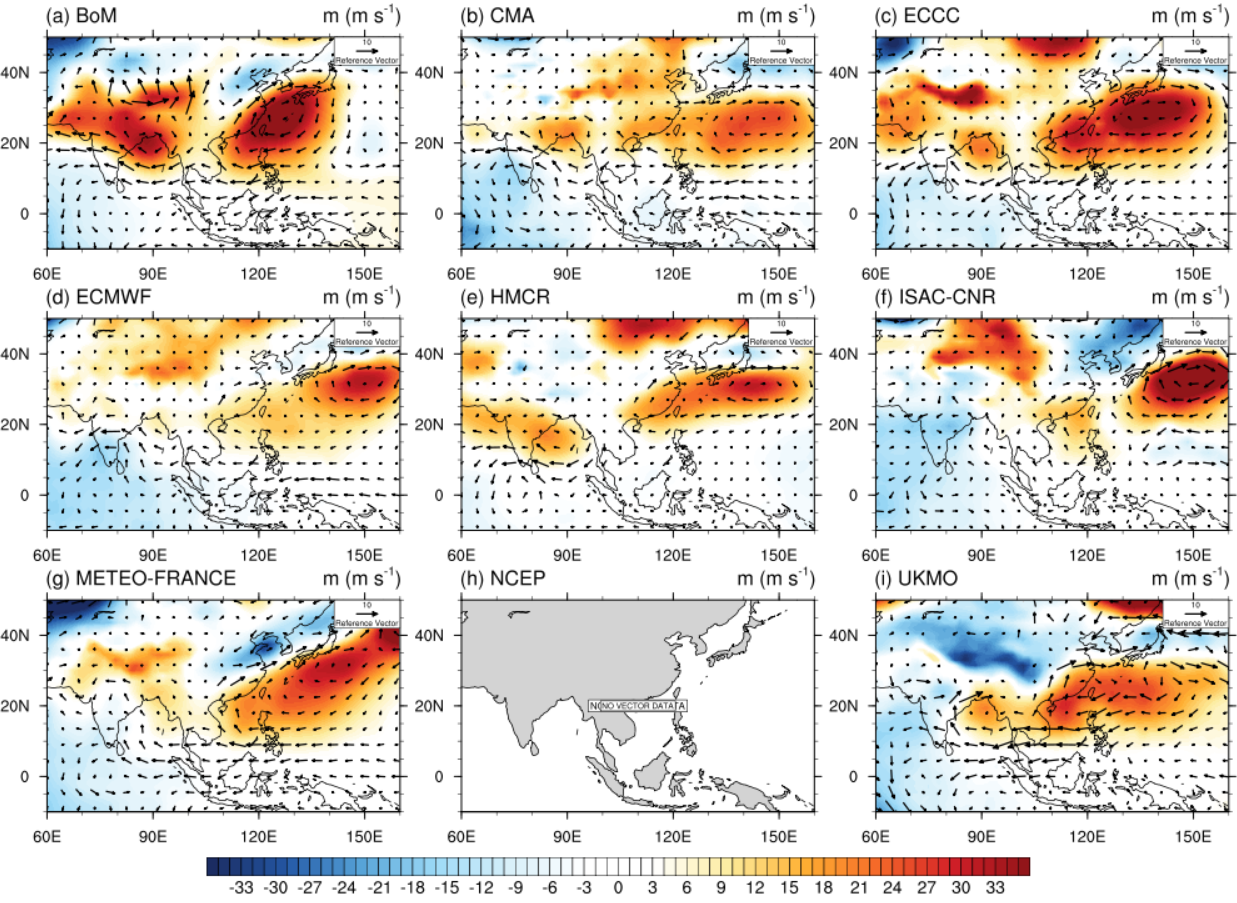


**Figure S12.** Same as Fig. S11, but for the initialization date June 25, 2020. NCEP had no MJO active days.


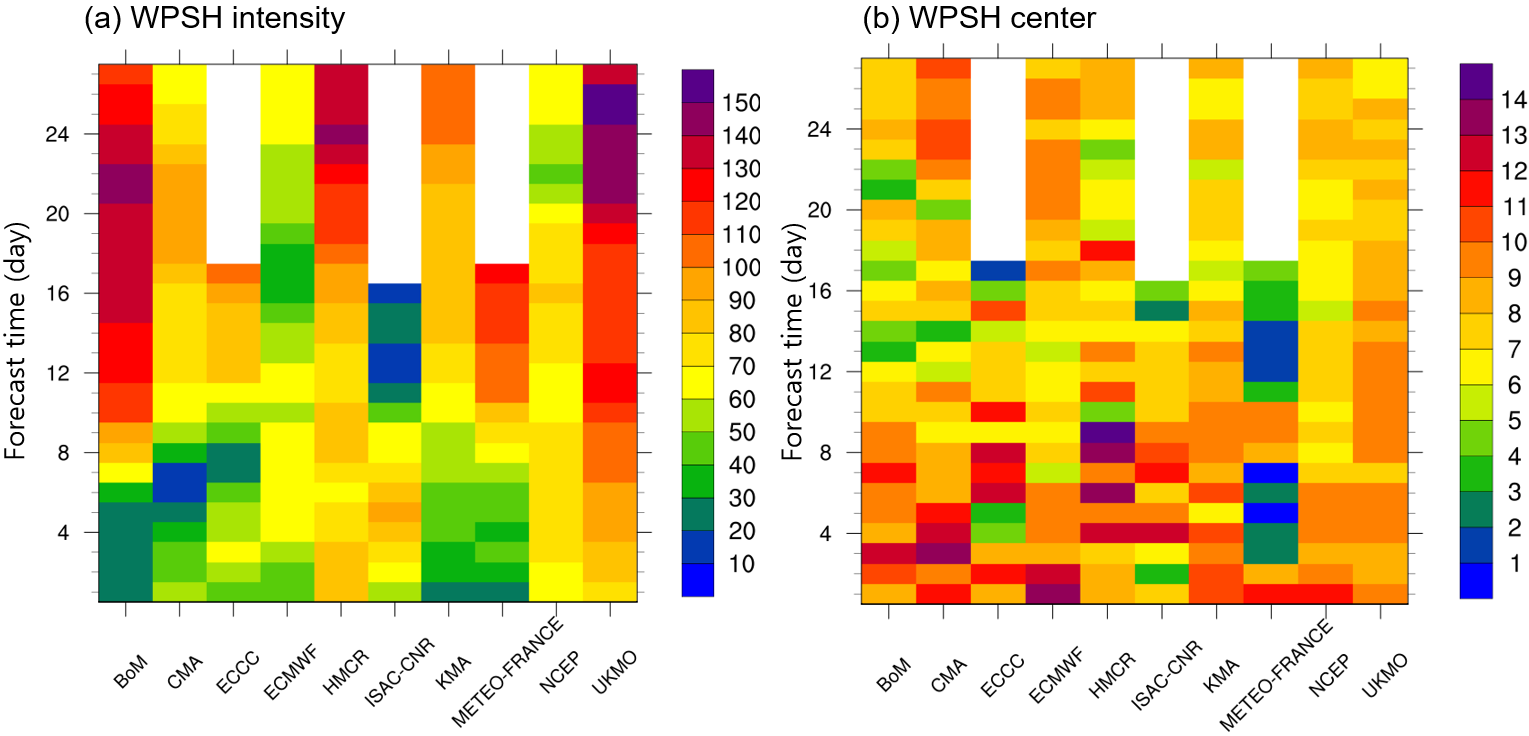


**Figure S13.** Error matrices of the Root Mean Squared Error (RMSE) of (a) intensity and (b) center of WPSH for the forecast time of 1-27 days averaged during the initial time from June 1 to July 31, 2020 in S2S models. White color indicates no value.
